# An improved radiosynthesis of [^18^F]FAraG, a PET radiotracer for imaging T‐cell activation

**DOI:** 10.1002/jlcr.3999

**Published:** 2022-09-05

**Authors:** Daniel P. Holt, Robert F. Dannals

**Affiliations:** ^1^ Division of Nuclear Medicine, Department of Radiology The Johns Hopkins University School of Medicine Baltimore Maryland USA

**Keywords:** cGMP, F‐18, FAraG, PET chemistry, radiosynthesis

## Abstract

In this concise practitioner protocol, the radiochemical synthesis of 2′‐deoxy‐2′‐[^18^F]fluoro‐9‐β‐d‐arabinofuranosylguanine ([^18^F]FAraG) suitable for human positron emission tomography (PET) studies is described and the results from validation productions are presented. The high specific activity (sometimes referred to as molar activity) radiotracer product is prepared as a sterile, apyrogenic solution that conforms to current Good Manufacturing Practice (cGMP) requirements established by the U.S. Food and Drug Administration.

## 1 INTRODUCTION

Over a decade ago, Namavari et al. reported the synthesis of 2′‐deoxy‐2′‐[^18^F]fluoro‐9‐β‐d‐arabinofuranosylguanine ([^18^F]FAraG) as a novel radiotracer for imaging T‐cell activation with positron emission tomography (PET).^1^ In the years that have followed, there have been reports of the radiotracer being used,^2^, ^3^, ^4^, ^5^, ^6^, ^7^ but no further report on substantive modifications to the radiotracer synthesis except for some mention of radiochemical yields and purities and a recent publication using solid phase extraction (SPE) for preclinical studies.^8^

This report describes an improved radiochemical synthesis of [^18^F]FAraG with full cGMP‐compliant quality control (QC) specifications and results. This procedure is suitable for human PET studies.

## 2 STANDARD REAGENT STATEMENT

**FIGURE 1:**
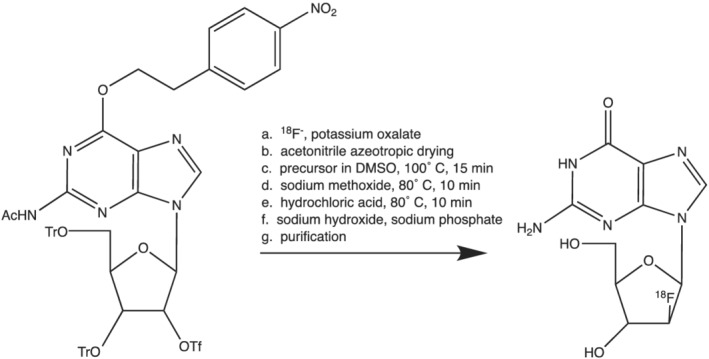
Synthesis of [^18^F]FAraG via nucleophilic radiofluorination

Reagents and solvents were obtained from Sigma‐Aldrich or Thermo Fisher Scientific in American Chemical Society (ACS) and high‐performance liquid chromatography (HPLC) grade, respectively, unless otherwise noted. The precursor (2‐*N*‐acetyl‐6‐*O*‐((4‐nitrophenyl)ethyl)‐9‐(3,5‐di‐*O*‐trityl‐2‐trifyl‐β‐d‐ribofuranyl)guanine) and the authentic standard (2′‐deoxy‐2′‐fluoro‐9‐β‐d‐arabinofuranosylguanine; Figure 1) were provided by CellSight, a sponsor of this research.

The semi‐preparative HPLC system consisted of an Agilent 1260 Prep pump with a VICI injector, a Knauer 200 UV detector (254 nm), and a Bioscan Hot Cell interface with a diode radioactivity detector. The semi‐preparative HPLC column was a Waters Atlantis T3 C18 5 μm 10 × 250 mm column eluted with a mixture of 3% ethanol (Pharmco‐Aaper, CT): 97% phosphate‐buffered saline at pH 7.4 at flow rate of 6 ml/min. The analytical chromatography system included an Agilent 1260 Infinity II System equipped with a quaternary pump (G7111B), HiP ALS multisampler (G7167A), and diode array detector HS (G7117C) detector with a Max‐Light flow cell set to 254 nm as well as a Bioscan Flow‐Count interface with a Bioscan Flow‐Count NaI radioactivity detector. The analytical HPLC was performed on a Waters Atlantis T3 C18 5 μm 4.6 × 150 mm column eluted with a mixture of 3% acetonitrile:97% 10 mM sodium dihydrogen phosphate at a flow rate of 2 ml/min. Chromatographic data were acquired and analyzed with Agilent OpenLAB CDS EZChrom Edition chromatography (Rev. A.04.09).

Radioactivity measurements were made using a Comecer model IBC‐LITE dose calibrator (Castel Bolognese, Italy) with Veenstra VIK‐202/203 ionization chambers (Joure, the Netherlands).

Residual solvent levels were analyzed using an Agilent 7890A gas chromatograph (GC) with Agilent 7693A automatic liquid sampler with data acquisition using Agilent OpenLAB CDS EZChrom Edition chromatography (Rev. A.04.09). The analysis was performed on an Agilent J&W DB‐WAX (polyethylene glycol phase: U.S. Pharmacopeia [USP] G16 and G20) 30 m, 0.25 mm ID, 0.25 μm film column connected to split–splitless inlet with its temperature set at 200°C, split ratio set at 50:1, and FID detector set to 300°C.

Endotoxin testing was performed on a Charles River Laboratories Endosafe® nexgen‐PTS (Wilmington, MA).

Sterility testing was conducted in aerobic and anaerobic media according to USP <71>.

The radionuclidic purity was examined with an AccuSync (Milford, CT) SA1000‐1S 1024‐channel spectrum analyzer.

## 3 SYNTHESIS PROCEDURE

### 3.1 Production of [^18^F]fluoride

[^18^O]Water (98%, Huayi Isotopes [Shanghai, China], ~1.7 ml) was loaded into a niobium‐body, high‐yield [^18^F]fluoride target on a General Electric Medical Systems PETtrace cyclotron (GEMS; Waukesha, WI). The target was bombarded with a 16 MeV proton beam of 60 μA for up to 30 min to produce at least 1.5 Ci (37 GBq) of aqueous [^18^F]fluoride via the ^18^O(*p*,*n*)^18^F nuclear reaction.

### 3.2 Preparation of the radiofluorination module and solid‐phase extraction system

The radiochemical synthesis of [^18^F]FAraG was performed on a custom‐made, nucleophilic radiofluorination module as described previously (see Figure S1).^9^ The setup of this module involved attaching a Chromafix 30‐PS‐HCO_3_ SPE resin cartridge (ABX GmbH, Germany) cartridge for trapping cyclotron‐produced [^18^F]fluoride. The resin elution and azeotropic drying reagents were connected in vented vials (2 ml, Agilent) containing a solution of potassium oxalate (9.5 mg) and Kryptofix® 222 (40 mg) in 50% aqueous acetonitrile (600 μl) and 4 ml vial with acetonitrile for azeotropic drying. A vial of precursor (4.5 mg) in dimethylsulfoxide (400 μl, DMSO) was connected. The chemicals for performing the deprotection of the radiolabeled intermediate were connected in vials: sodium methoxide (200 μl, 0.5 M in methanol), 2 N hydrochloric acid (500 μl), and 1 N sodium hydroxide (1.2 ml). A vial containing sodium dihydrogen phosphate (550 mg in 2 ml of water) was connected to the module. Nitrogen gas (UHP, Matheson Gas, MD) was used for evaporation and transfer of all solutions.

### 3.3 Azeotropic drying of [^18^F]fluoride

After the preparation of the radiofluorination module, [^18^F]fluoride was delivered via PEEK tubing to the receiving vessel as a solution of [^18^O]water where it was assayed for radioactivity content. The recovery of [^18^F]fluoride was performed by passing the radioactive target solution through a Chromafix 30‐PS‐HCO3 anion‐exchange cartridge (Macherey‐Nagel, Germany), and the [^18^O]water was collected for recycling. The [^18^F]fluoride was then eluted with an aliquot of potassium oxalate/Kryptofix® 222 (K222) solution (200 μl of abovementioned solution) using a Tecan Cavro® syringe pump (Switzerland). The eluted radioactivity was flushed to the reaction vial with an additional 250 μl of acetonitrile followed by a 1 ml air push from the syringe pump. The second step was a further enhancement of the chemical reactivity of K/K222^+^/[^18^F]F^−^ by azeotropic drying, which took place in the reaction vial. The vial was heated to 110°C with nitrogen flow (600 ml/min) during which two aliquots of acetonitrile (250 μl) were added permitting the acetonitrile–water mixture to evaporate to dryness.

### 3.4 Synthesis of [^18^F]FAraG

The solution of the FAraG precursor in anhydrous DMSO was added into the reaction vial, and the vial was heated to 100°C for 15 min and then cooled to 60°C. Sodium methoxide was then added followed by heating at 80°C for 10 min, after which the solution was cooled to 60°C. Hydrochloric acid was added next, and heating was continued at 80°C for 10 min, after which the solution was cooled to 60°C. Sodium hydroxide was added to assist adjust the pH of the reaction mixture towards neutrality. Lastly, the solution of sodium dihydrogen phosphate was added to dilute the reaction mixture for HPLC purification.

### 3.5 Purification and formulation of [^18^F]FAraG

**FIGURE 2:**
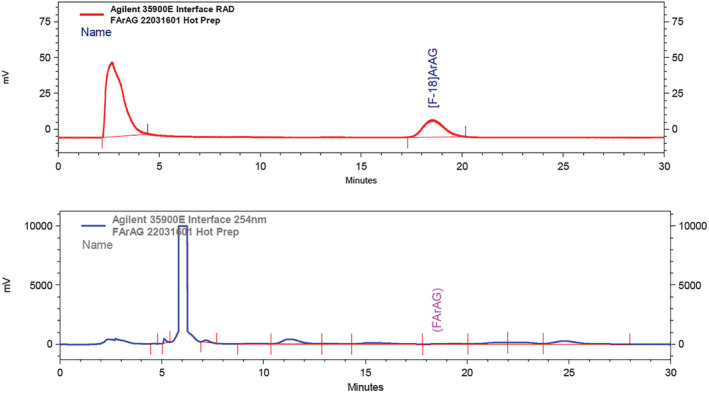
Preparative chromatogram of [^18^F]FAraG (red—radioactivity; blue—UV at 245 nm)

Purification of the crude [^18^F]FAraG reaction mixture was accomplished by semi‐preparative HPLC. The product had a retention time of ~18 min (see Figure 2). The [^18^F]FAraG product was directly collected from the HPLC through a sterilizing 0.22 μm Millex FG filter (Millipore, MA) into a sterile, pyrogen‐free final product vial (Huayl) prefilled with 4 ml sterile 0.9% sodium chloride for injection. This concluded the radiochemical synthesis, and the final product was submitted for QC testing. In the three validation runs and subsequent nine production runs reported here (Table S1), [^18^F]FAraG was isolated with an average radiochemical yield of 8.0 ± 1.6% (not corrected for decay, *n* = 12) from starting an average of 52.8 GBq (1426 mCi) [^18^F]fluoride, with an average time of synthesis of 97 min, and an average specific radioactivity (also known as molar radioactivity) of 568 ± 532 GBq/μmol (15,359 ± 14,375 mCi/μmol).

## 4 QUALITY CONTROL PROCEDURES

QC testing including visual inspection, radiochemical identity, radiochemical purity, specific activity calculation, residual solvent analysis, pH measurements, residual K222 analysis, filter integrity testing, radionuclide identity by half‐life measurement and multichannel spectral analysis, endotoxin analysis, and sterility testing have all been described previously^10^ and are not repeated here.

The QC procedures performed based upon current requirements for radiotracers set forth in the USP^11^ for three repeat validation batches of [^18^F]FAraG produced according to the described method are summarized in Table 1. Each of the three batches met all established QC criteria. Additional QC data for the subsequent batches are shown in the supporting information.

**TABLE 1:** Release and stability test data for three qualification batches of [^18^F]FAraG injection manufactured at the JHU PET center

[^18^F]FAraG batch number		FAraG Validation 1	FAraG Validation 2	FAraG Validation 3
Test	Specification			
Initial appearance	Clear, colorless solution, no visible particulate matter	Conforms	Conforms	Conforms
Appearance 240 min after EOS	Clear, colorless solution, no visible particulate matter	Conforms	Conforms	Conforms
Initial radiochemical purity, % (*t* = 0 min)	≥90%	95.2%	95.7%	97.1%
Expiry radiochemical purity, % (*t* = 240 min)	≥90%	96.1%	96.4%	97.0%
pH, initial	6.5–8.5	7.5	7.5	7.5
pH, expiry	6.5–8.5	7.5	7.5	7.5
Chemical purity	All others	FAraG: 0.16 μg/ml	FAraG: 0.24 μg/ml	FAraG: 0.07 μg/ml
	≤2 μg/ml	Others: 0.13 μg/ml	Others: 0.22 μg/ml	Others: 0.08 μg/ml
Yield	>20 mCi [^18^F]FAraG (referenced to assay recorded at end of filtration)	116.4 mCi	210 mCi	104.8 mCi
Specific activity	>1000 mCi/μmol of [^18^F]FAraG (referenced to end of filtration)	11,893 mCi/μmol	14,033 mCi/μmol	28,800 mCi/μmol
Identity (HPLC)	HPLC retention time is within 10% of reference standard	1.38%	1.34%	1.75%
Radionuclidic purity	T1/2 Calc = 105–115 min	110.13 min	110.63 min	111.69 min
Bubble point	>13 psi	16 psi	15 psi	15 psi
Kryptofix analysis	<50 μg/ml	Conforms	Conforms	Conforms
Residual solvent analysis (GC)	Ethanol ≤10%	3% ethanol	3% ethanol	3% ethanol
	Acetonitrile ≤273 ppm	24 ppm MeCN	65 ppm MeCN	46 ppm MeCN
	DMSO ≤ 3333 ppm	109 ppm DMSO	244 ppm DMSO	67 ppm DMSO
	Methanol ≤2045 ppm	9 ppm methanol	10 ppm methanol	2 ppm methanol
Bacterial endotoxin	<11 USP EU/ml	<5 EU/ml	<5 EU/ml	<5 EU/ml
Sterility	No growth observed	Conforms	Conforms	Conforms

**FIGURE 3:**
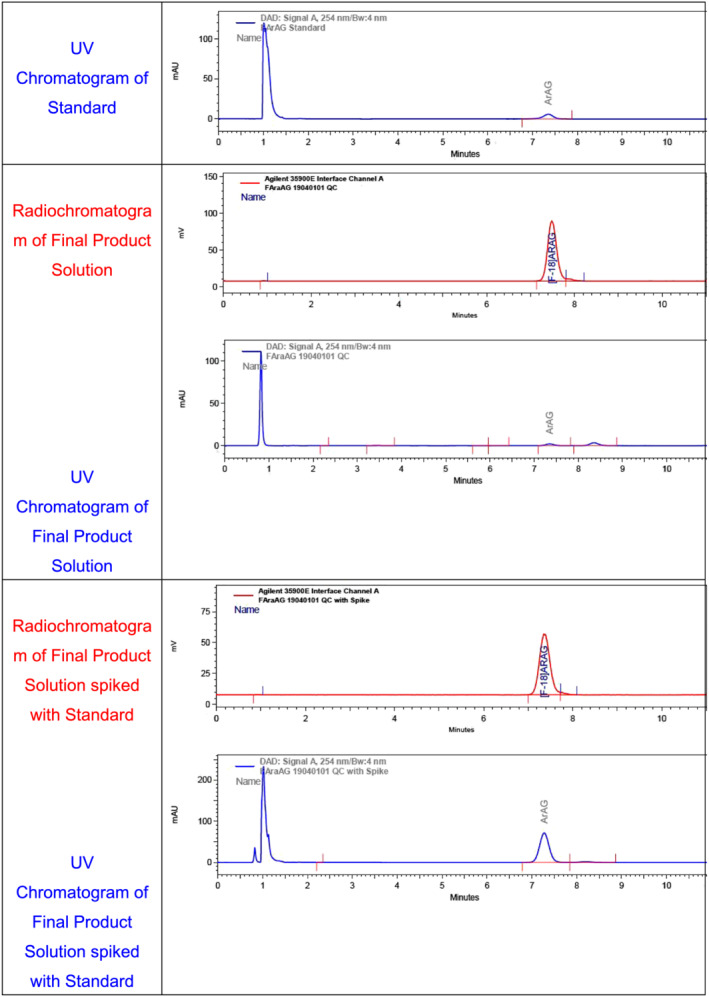
Quality control chromatograms of [^18^F]FAraG

Figure 3 shows typical analytical chromatograms observed during the determination of the radiochemical identity, radiochemical purity, and specific activity. This includes a standard of the authentic nonradioactive product to establish system suitability, the final radiotracer product, and a co‐injection of authentic FAraG with the radiotracer product.

## 5 DISCUSSION

The original publication for [^18^F]FAraG from 2011 described a synthesis that took between 140 and 160 min and provided this radiotracer in a 7% to 10% (decay‐corrected) yield with a specific activity of 30–48 GBq/μmol (800–1300 mCi/μmol). Given the lengthy synthesis that involved an intermediate purification by HPLC, deprotection, then a second HPLC to isolate the final product, the 7% to 10% yield that was decay corrected is calculated to be ~2.5–4% at the end of synthesis (EOS), which would be the time of its use, after suitable QC. In a subsequent publication, a decay‐corrected yield of 2–5% with higher specific activity (111–296 GBq/μmol; 3000 to 8000 mCi/μmol with no reference to its time of measurement) and a synthesis time of 95 min was reported.^4^ In the same year, very similar results were presented.^5^ On average, the non‐decay‐corrected radiochemical yields were all between 2% and 5% from these previous studies.

In the work presented here for the three validation runs and nine subsequent production runs, the average time of synthesis was 97 min from the time the target was emptied and [^18^F]fluoride was trapped on the anionic exchange cartridge until the final product was assayed for its radioactivity content at the EOS. The non‐decay‐corrected radiochemical yield at EOS was 6.0–11.4% (average 8.0%), and the specific radioactivity calculated at EOS was 337–1066 GBq/μmol (average 568 GBq/μmol) (9117–28,800 mCi/μmol; average 15,359 mCi/μmol). The final product radiochemical purity was an average of 97.9% at EOS, and the product remained stable at room temperature for at least 240 min. No production failures were observed due to radiochemistry or final product QC issues.

Based on our initial workup of the published synthesis, the precursor did not appear to be stable when the originally reported reagent amounts of K222 and potassium carbonate were used during the radiofluorination step. Thus, alternative conditions were sought. To attempt to increase the fluorination yield and preserve the precursor, the following bases were investigated: tetrabutylammonium bicarbonate, K222/potassium bicarbonate, K222/potassium oxalate, and K222/potassium acetate. No radiofluorination was observed when tetrabutylammonium bicarbonate was used. Potassium bicarbonate, acetate, and oxalate all provided higher radiofluorination yields than the original potassium carbonate (data not shown). In the developmental workup, the use of potassium oxalate produced the highest radiofluorination yield, and it was selected for radiotracer validation syntheses.

The near doubling of the radiochemical yield was achieved by optimizing individual steps in the previously reported synthesis. The synthesis used potassium oxalate instead of potassium carbonate and heated the precursor/fluoride reaction mixture at 100°C for 15 min (compared to 85°C for 45 min in the original publication). By eliminating the intermediate HPLC purification and changing the preparative HPLC mobile phase to a mixture of ethanol and phosphate‐buffered saline at pH 7.4, the radiotracer could be collected directly off the semi‐preparative HPLC column through a sterilizing microfilter into the final product vial, which eliminated the evaporation under reduced pressure step and reformulation from the original paper.

It is noted that the final product does show some additional UV peaks in the QC chromatogram (Figure 2). These peaks are accentuated by the small carrier mass of the final product, [^19^F]FAraG. At a lower specific radioactivity, it is quite possible that these might be considered too small to integrate. Lacking a regulatory definition for chemical purity for PET radiotracers, the chemical purity specification of less than 2 μg/ml has been used for this radiotracer for all non‐product (or “all other”) UV‐absorbing peaks shown in the QC chromatogram. This limit was determined based on the amounts of starting materials and the target goal of eliminating 99.5% of those starting materials from the final product solution.

## 6 CONCLUSION

In summary, a cGMP protocol for the radiosynthesis of [^18^F]FAraG, a radioligand for imaging soluble epoxide hydrolase, has been developed. Although the custom radiofluorination module used in this work has been developed to limit contamination of extraneous sources of carrier fluoride ion and permit reactions at higher pressures, it is likely that commercial radiofluorination synthesis devices should be able to adapt the procedure described here with potentially higher overall radiotracer product yields. The highly reproducible radiochemical synthesis produced sufficient quantities of [^18^F]FAraG at high specific radioactivity and chemical and radiochemical purity for use in human PET studies.

## Supporting information


**Data S1:** Supporting information
