# Supplementary material for: An improved radiosynthesis of [18F]FAraG, a PET radiotracer for imaging T‐cell activation
Source: J Labelled Comp Radiopharm. 2022 Sep 5;65(12):302–8. doi: 10.1002/jlcr.3999 (PMC9826029; doi:10.1002/jlcr.3999)
Supplement: Supplementary file 1 — Data S1: Supporting information [file JLCR-65-302-s001.docx]

**SUPPLEMENTAL DATA:**

**An Improved Radiosynthesis of [^18^F]FAraG,**

**a PET Radiotracer for Imaging T-Cell Activation**

Daniel P. Holt and Robert F. Dannals*

*Division of Nuclear Medicine, Department of Radiology*

*The Johns Hopkins University School of Medicine*

*600 North Wolfe Street, Nelson B1-152*

*Baltimore, Maryland 21287 USA*

** - author for correspondence <rfd@jhu.edu>*

The determination of the specific activity (molar activity) of FAraG was determined by analytical HPLC against a 7 level, 6 replicate each, calibration curve spanning a range of 5.6 pmoles up to 358 pmoles. The calibration curve had a goodness of fit (r^2^) of 0.999999.


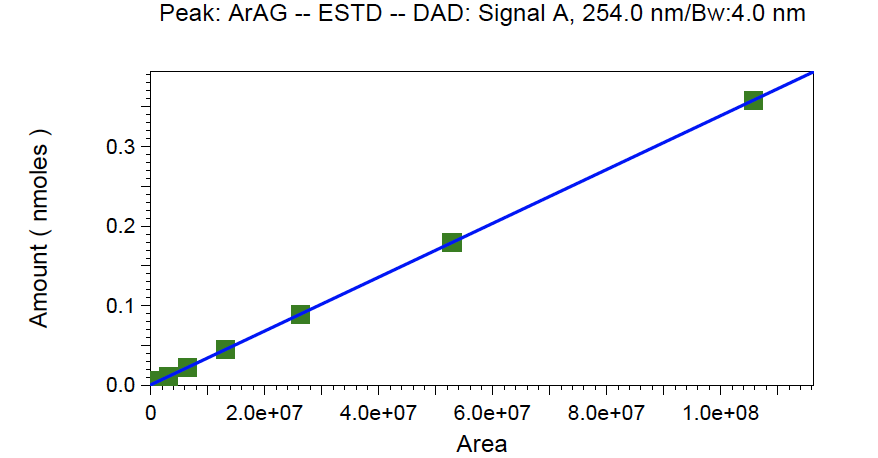


Analytical chromatography system:

Agilent 1260 Infinity II System equipped with a quaternary pump (G7111B)

HiP ALS multisampler (G7167A)

Diode array detector HS (G7117C) detector with Max-Light flow cell set to 254 nm.

Agilent OpenLAB CDS EZChrom Edition chromatography (Rev. A.04.09).

The analytical HPLC Conditions:

Waters Atlantis T3 C18, 5µ, 4.6 x 150 mm column

Mobile phase: 3% acetonitrile : 97% 10 mM sodium dihydrogen phosphate

Flow rate: 2 mL/min.

The custom-built, nucleophilic radiofluorination module (RFM) used for the [^18^F]FAraG synthesis is 4th version RFM referenced by Hayden T. Ravert, Daniel P. Holt, and Robert F. Dannals. "A microwave radiosynthesis of the 4‐[18F]‐fluorobenzyltriphenylphosphonium ion." J Label Compd Radiopharm 57.12 (2014): 695-698. The system is a semi-automated radiosynthesis module built around standard off the shelf components, including VICI valves and Tecan Centris Cavro pumps. The reactions are done in Wheaton 5 mL V-vials or CEM Corporation microwave vials using a custom multi-port cap. The RFM is controlled by custom programming utilizing National Instruments LabView and LabView Real-time software.


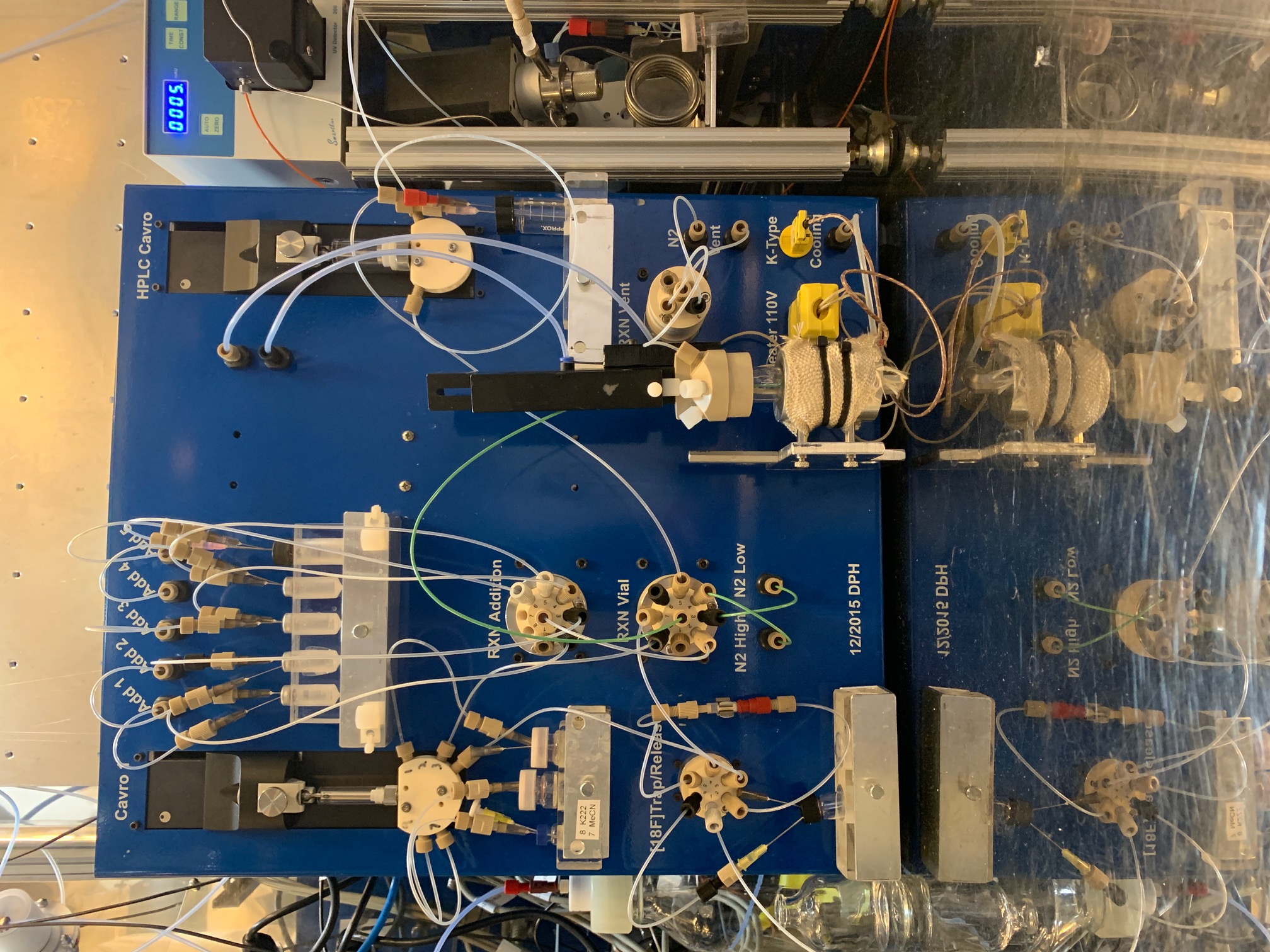


Figure 1.

Supplemental Table 1: Release test data from subsequent nine production batches of [^18^F]FAraG used for PET studies.

| Run Number: | 1 | 2 | 3 | 4 | 5 | 6 | 7 | 8 | 9 |
| --- | --- | --- | --- | --- | --- | --- | --- | --- | --- |
| Appearance: | Conforms | Conforms | Conforms | Conforms | Conforms | Conforms | Conforms | Conforms | Conforms |
| Radiochemical purity (%): | 99.4 | 97.4 | 100 | 97.6 | 99.7 | 98.1 | 97.7 | 99.8 | 97.6 |
| pH: | 7.5 | 7.0 | 7.0 | 7.5 | 7.0 | 7.0 | 7.5 | 7.5 | 7.5 |
| Yield (mCi): | 99.4 | 139.9 | 118 | 106 | 123.1 | 133.1 | 118 | 94.3 | 72.5 |
| Specific Activity (mCi/µmole): | 26,074 | 18,870 | 11,494 | 10,030 | 18,870 | 23,500 | 17,124 | 13,866 | 9,117 |
| HPLC Identity: | 0.80% | 2% | 1.20% | 2.19% | 2.25% | 2.59% | 2.29% | 2.53% | 2.27% |
| Radionuclidic purity: (by half-life): | 105.62 | 109.09 | 110.01 | 109.43 | 109.09 | 113.12 | 109.35 | 110.35 | 106.49 |
| Bubble-point (psi): | 16 | 14 | 16 | 14 | 15 | 15 | 15 | 16 | 17 |
| Kryptofix analysis: | Conforms | Conforms | Conforms | Conforms | Conforms | Conforms | Conforms | Conforms | Conforms |
| Bacterial Endotoxin (EU/mL): | <5 | <5 | <5 | <5 | <5 | <5 | <5 | <5 | <5 |
| Sterility: | Conforms | Conforms | Conforms | Conforms | Conforms | Conforms | Conforms | Conforms | Conforms |
| Chemical purity |  |  |  |  |  |  |  |  |  |
| FAraG (µg/mL): | 0.003 | 0.14 | 0.19 | 0.19 | 0.14 | 0.13 | 0.13 | 0.14 | 0.16 |
| All Others (µg/mL): | 0.1 | 0.009 | 0.11 | 0.11 | 0.08 | 0.05 | 0.11 | 0.02 | 0.13 |
| GC Analysis |  |  |  |  |  |  |  |  |  |
| Ethanol %: | 3.2 | 2.63 | 2.65 | 3.32 | 2.5 | 2.92 | 2.93 | 2.91 | 2.75 |
| Acetonitrile (ppm): | 116 | 57 | 43.3 | 66.5 | 65.6 | 105.7 | 93.7 | 39.8 | 44.1 |
| DMSO (ppm): | 70.9 | 4.5 | 0 | 7 | 17.7 | 193.2 | 468 | 23.9 | 255.8 |
| Methanol (ppm): | 0 | 2 | 0 | 0 | 0 | 0 | 0 | 0 | 0 |
|  |  |  |  |  |  |  |  |  |  |
